# A rare case of small bowel leiomyosarcoma presenting with acute gastro-intestinal bleeding

**DOI:** 10.1259/bjrcr.20160089

**Published:** 2016-12-22

**Authors:** Christine Jacqueline Tolman

**Affiliations:** Department of Radiology, Medisch Centrum Haaglanden, The Hague, Netherlands

## Abstract

A 62-year-old Turkish female was admitted to our hospital with acute, progressive melena. Gastroscopy and colonoscopy could not reveal the cause of the melena. Subsequent CT angiography demonstrated a large, exophytic mass in the ileocecal junction as a source of the haemorrhage, leading to urgent laparotomy and resection. Histopathology revealed a low grade leiomyosarcoma (LMS) and confirmatory immunological staining. Primary LMS of the small bowel is an extremely rare gastrointestinal (GI) malignancy. Presentation with acute GI bleeding is even more exceptional, since LMS is a mainly intramural, exophytic tumour of the bowel wall. Immunohistochemistry plays a crucial role in differentiating LMS from GI stromal tumour. The work up of occult small bowel neoplasms currently consists of MRI enterography or enteroclysis and wireless capsule endoscopy. Treatment is surgical resection. This case highlights the non-specific imaging features of ileal LMS and highlights the management of acute GI bleed.

## Clinical presentation

A 62-year-old Turkish female with a medical history of caesarean section, second degree atrioventricular block treated with a pacemaker and chronic paroxysmal atrium fibrillation treated with fenprocoumon (anticoagulant), presented in the Emergency Room with one day of rectal blood loss. No history of previous gastrointestinal complaints. On clinical examination, patient had a non-tender abdomen. Rectal examination showed dried blood around the anus, no palpable mass and no melena. Patient had a tight tension of 100/60 mmHg, which was 115/70 mmHg after 1 l filling with sodium chloride infusion. Laboratory results showed an International Normalized Ratio (INR) of 2.0 and haemoglobin of 4.9.

**Figure 1.**
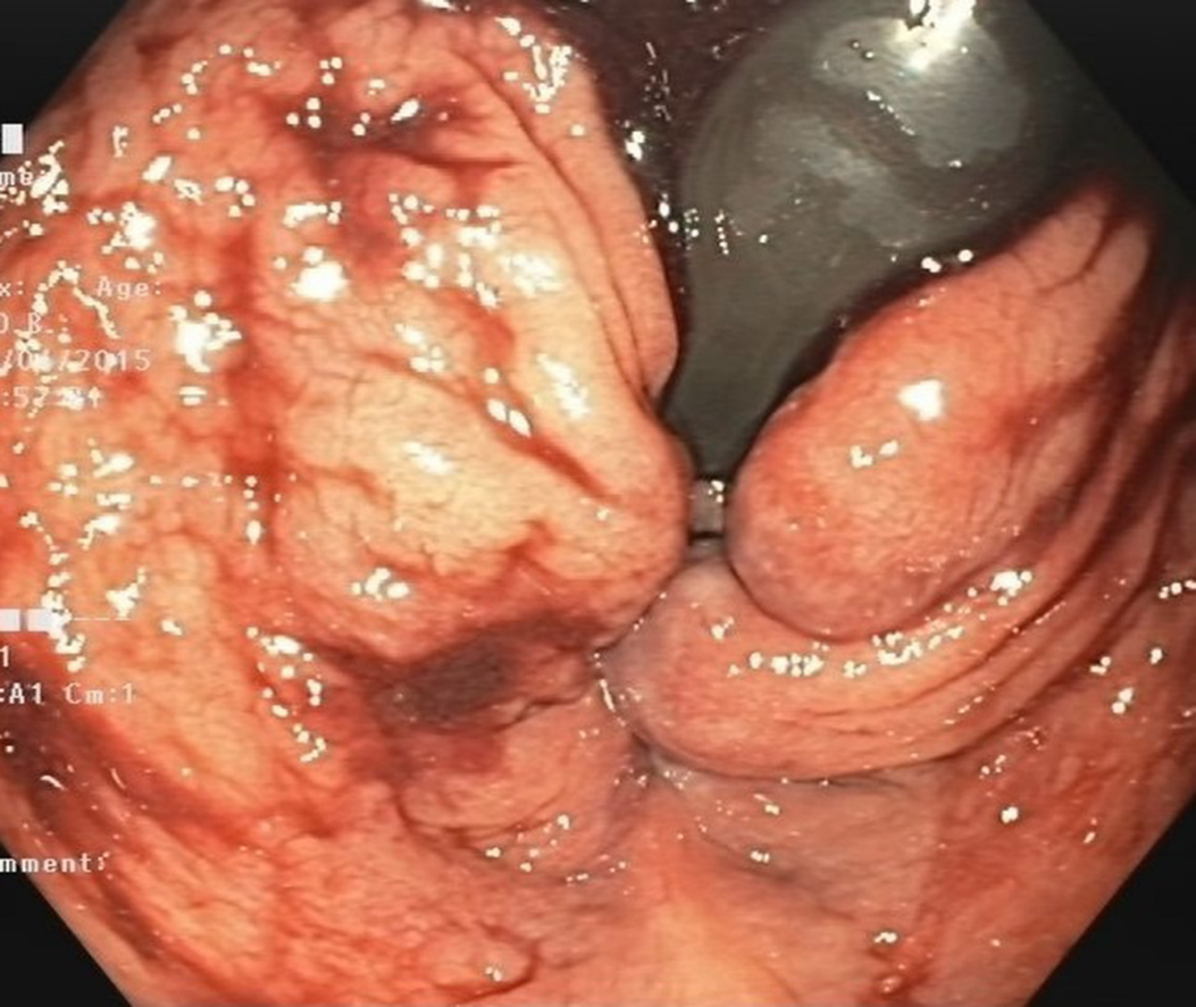
Push gastroenteroscopy shows a vast haemorrhage in colon and terminal ileum, without establishment of the source of bleeding.

Patient underwent immediate esophagogastroscopy, which showed no focus of bleeding from stomach into descending part of duodenum. Push gastroenteroscopy (using a larger endoscope to examine a large part of the small intestine, but still with limited reach to the (entire) ileum) showed much blood in colon and terminal ileum (Figure 1), suggesting a bleeding the distal jejunum or ileum. No bleeding focus could be established, also after investigation with water colonoscopy deep in the jejunum. After multiple erythrocyte transfusions the patients’ rectal bleed persisted and her haemoglobin still declined to finally 3.1. A bleeding source in ileum or jejunum was still suspected and CT angiography (CTA) was performed.

## Imaging findings

**Figure 2.**
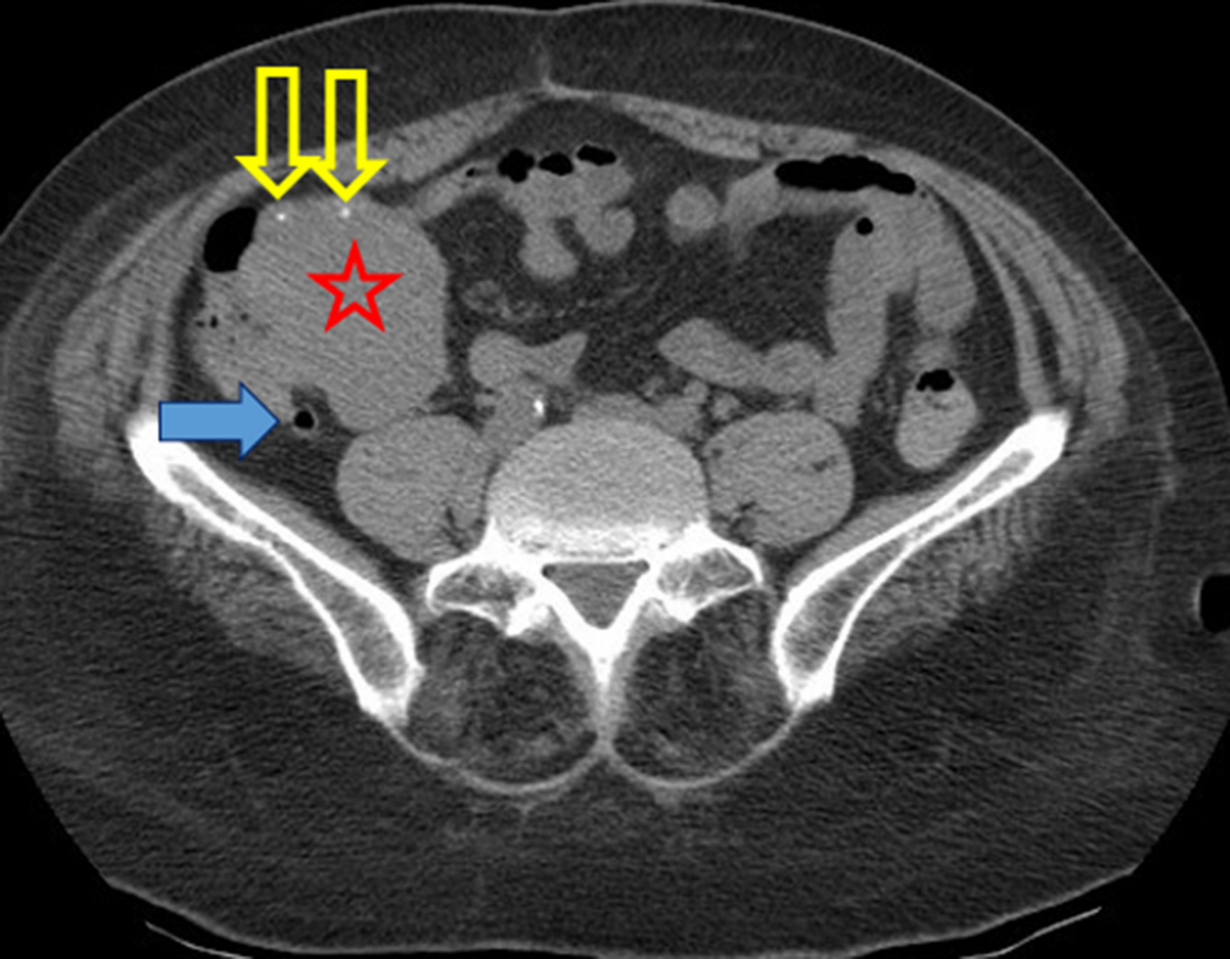
Axial non-enhanced CT scan. Large, isodense lobulated mass (red star) at the ileocecal junction with some small calcifications (open yellow arrows). Notice the normal air-filled appendix (blue arrow).

**Figure 3.**
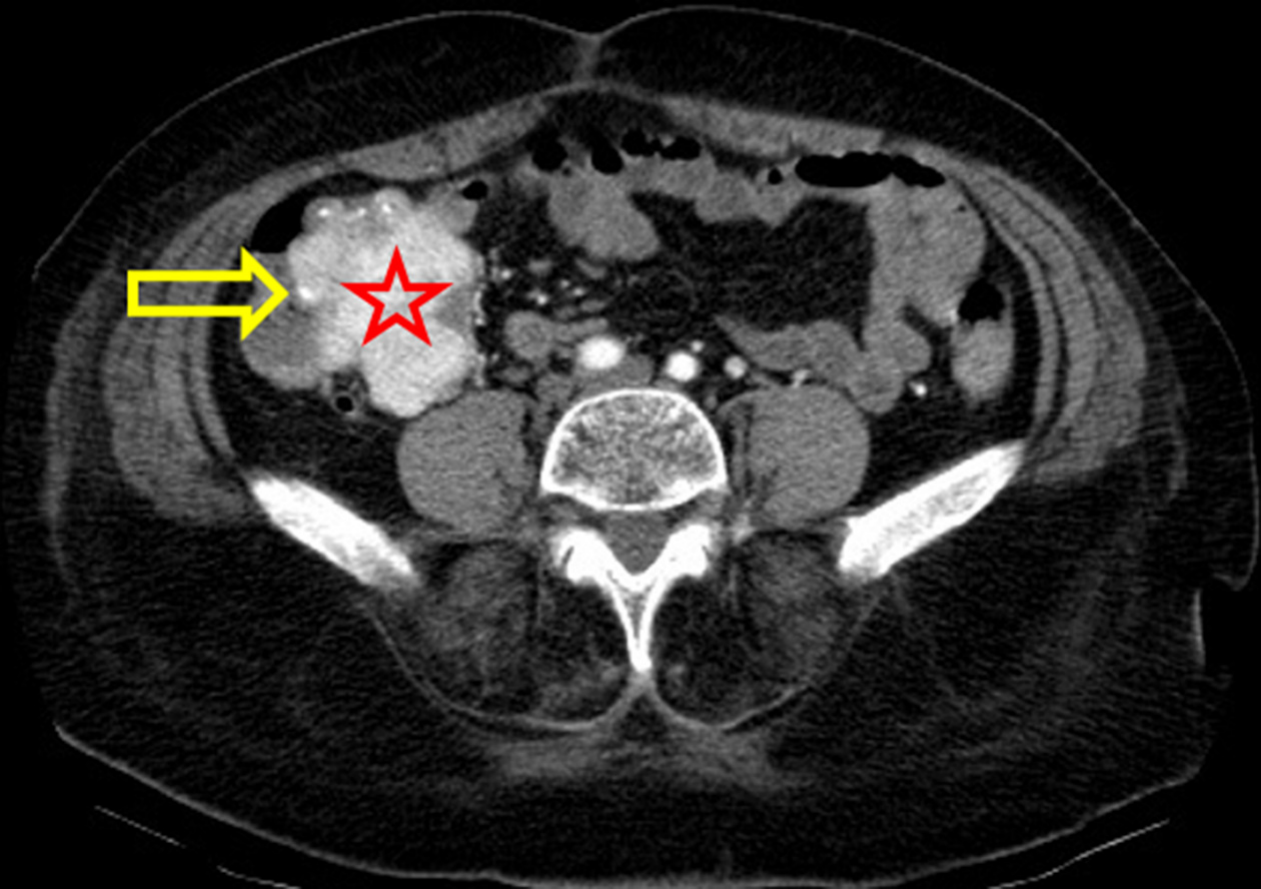
Axial enhanced CT scan, arterial phase. Heterogeneous enhancing mass (red star) in the wall of terminal ileum. Small jet of active contrast extravasation in the lumen of the cecum (yellow arrow).

**Figure 4.**
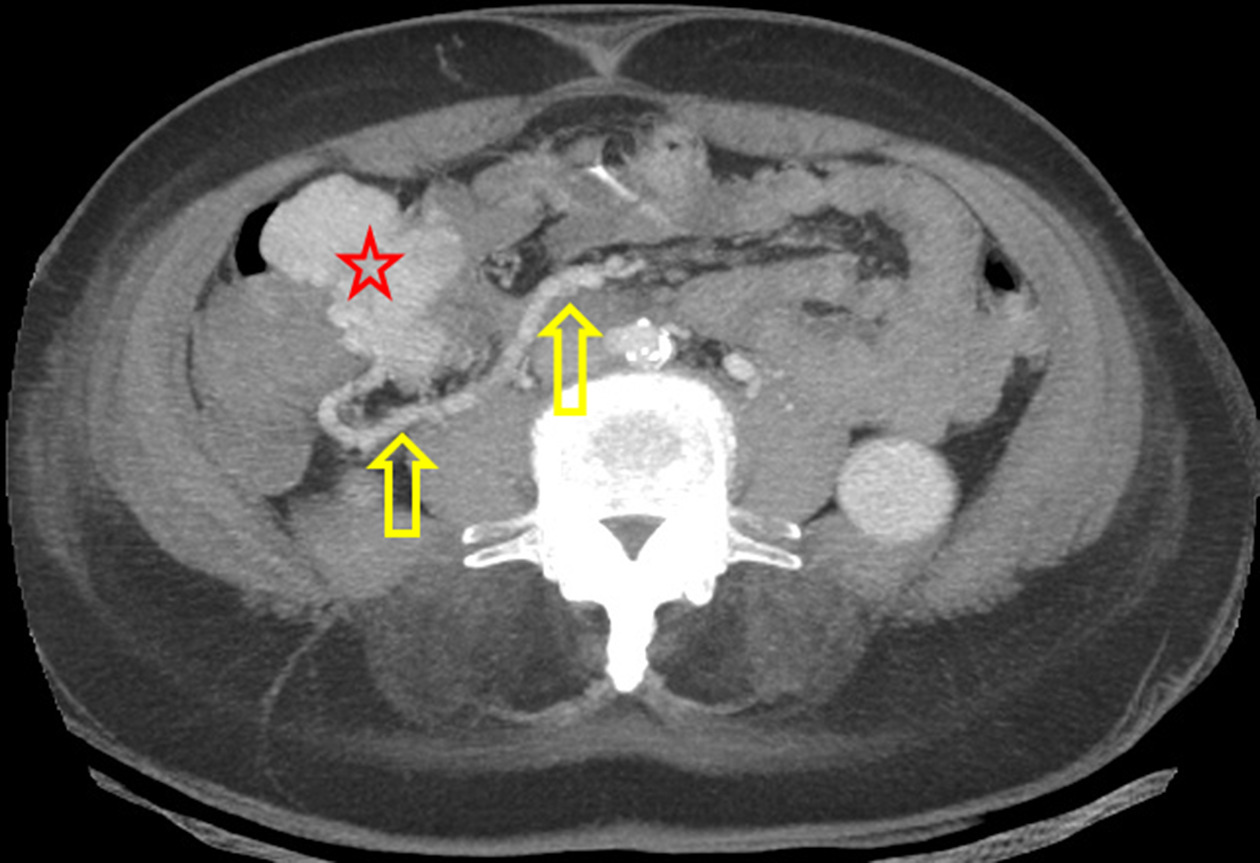
Maximum intensity projection of axial contrast-enhanced CT scan in arterial phase. Exophytic ileal mass (red star and drained by aberrant hypertrophic vascular structure originating from the portal vein (yellow arrows).

**Figure 5.**
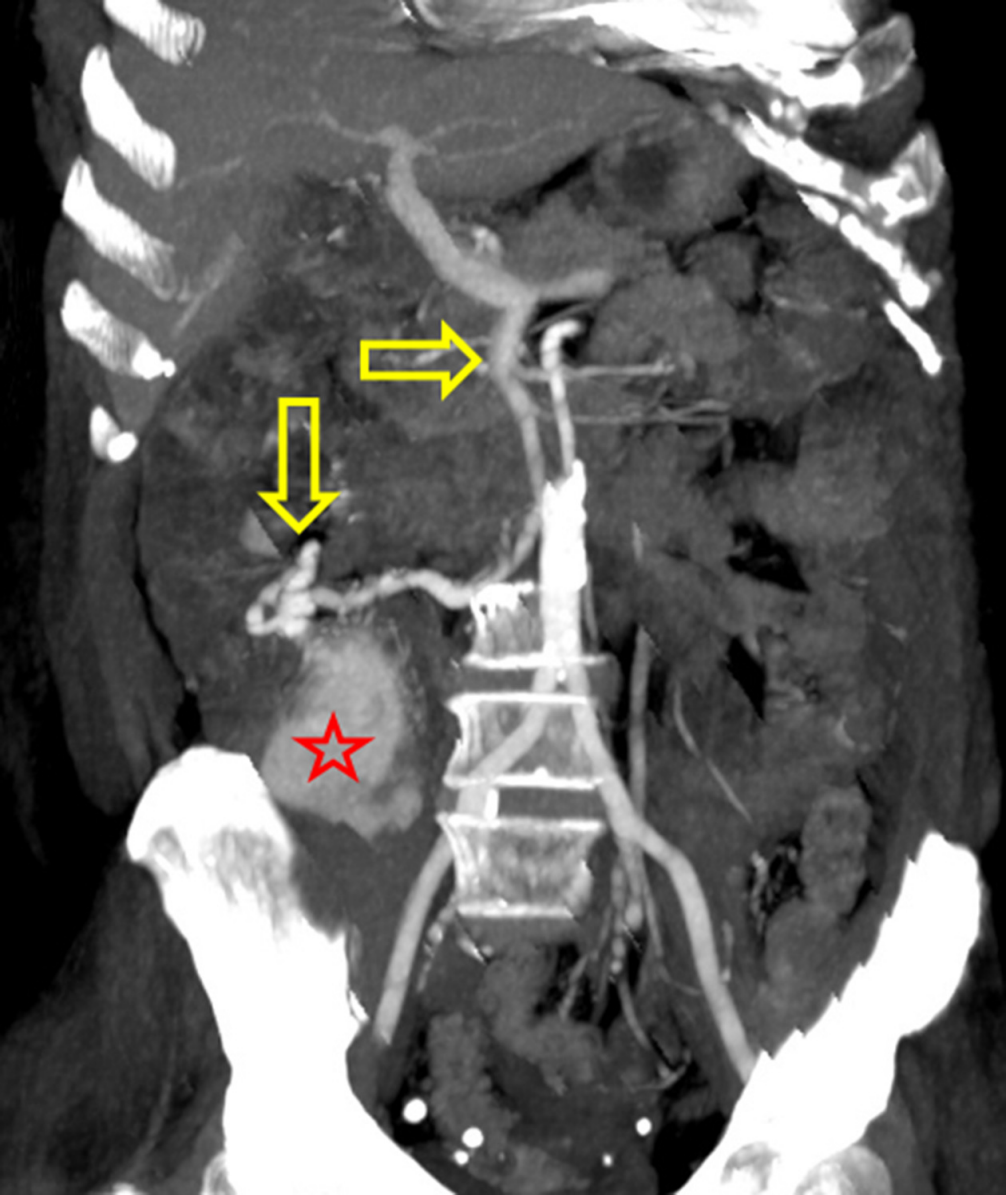
Maximum intensity projection of coronal contrast-enhanced CT scan in arterial phase. Exophytic ileal mass (red star), drained by aberrant hypertrophic vascular structure originating from the portal vein (yellow arrows).

A three-phase CTA of the abdomen (non-contrast, contrast-enhanced arterial and portal venous phase) was performed. Non-enhanced CT revealed an enlarged cecum and terminal ileum, with an exophytic, isodense mass with some calcifications mainly located in the wall of the terminal ileum (Figure 2). Contrast-enhanced CT (CECT) showed a small jet of active contrast extravasation in the lumen of the cecum and heterogeneous enhancement of the mass (Figure 3). An aberrant hypertrophic vascular structure drained from the confluence of superior mesenteric vein and portal vein to the mass, not to be mistaken with the normal appendix nor ileum (Figures 4 and 5). This finding at the time indicated the possibility of an arteriovenous malformation; however, no dominant arterial feeding vessel could be found on CTA and the large, hyperattenuating mass suggested an underlying neoplasm. There was no small bowel obstruction, no infiltration of perifocal mesenterial fat, no lymphadenopathy and no intraperitoneal free fluid. The liver and peritoneum appeared normal.

## Outcome

**Figure 6.**
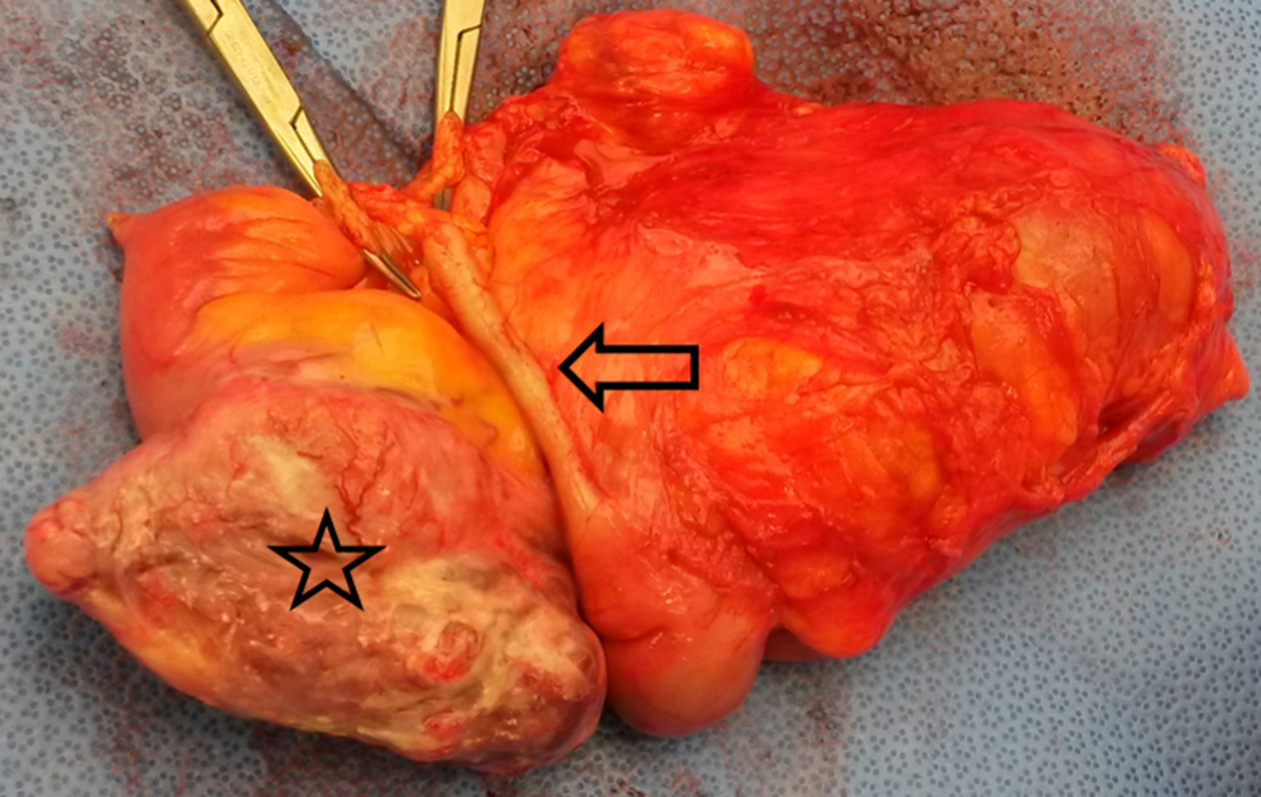
Gross photograph of ileocecal mass (star) during surgery, fed by an aberrant hypertrophic vein (arrow).

The main radiological differential diagnosis at the time an arteriovenous malformation, or a small bowel malignancy, either gastrointestinal stromal tumour or leiomyoma. No angiography with embolisation was performed since no arterial feeding vessel could be identified on CTA. The patient underwent surgery in acute setting since blood loss could not be controlled. During surgery an exophytic, hard palpable tumour was found in the wall of the terminal ileum. Ileocecal resection was performed with a side-to-side primary anastomosis with staplers. Also, the aberrant vein was identified and resected (Figure 6). There were no perioperative complications or during admission. The patient could be discharged in good clinical condition. Post-surgery follow-up remains unremarkable.

## Histopathology findings

**Figure 7.**
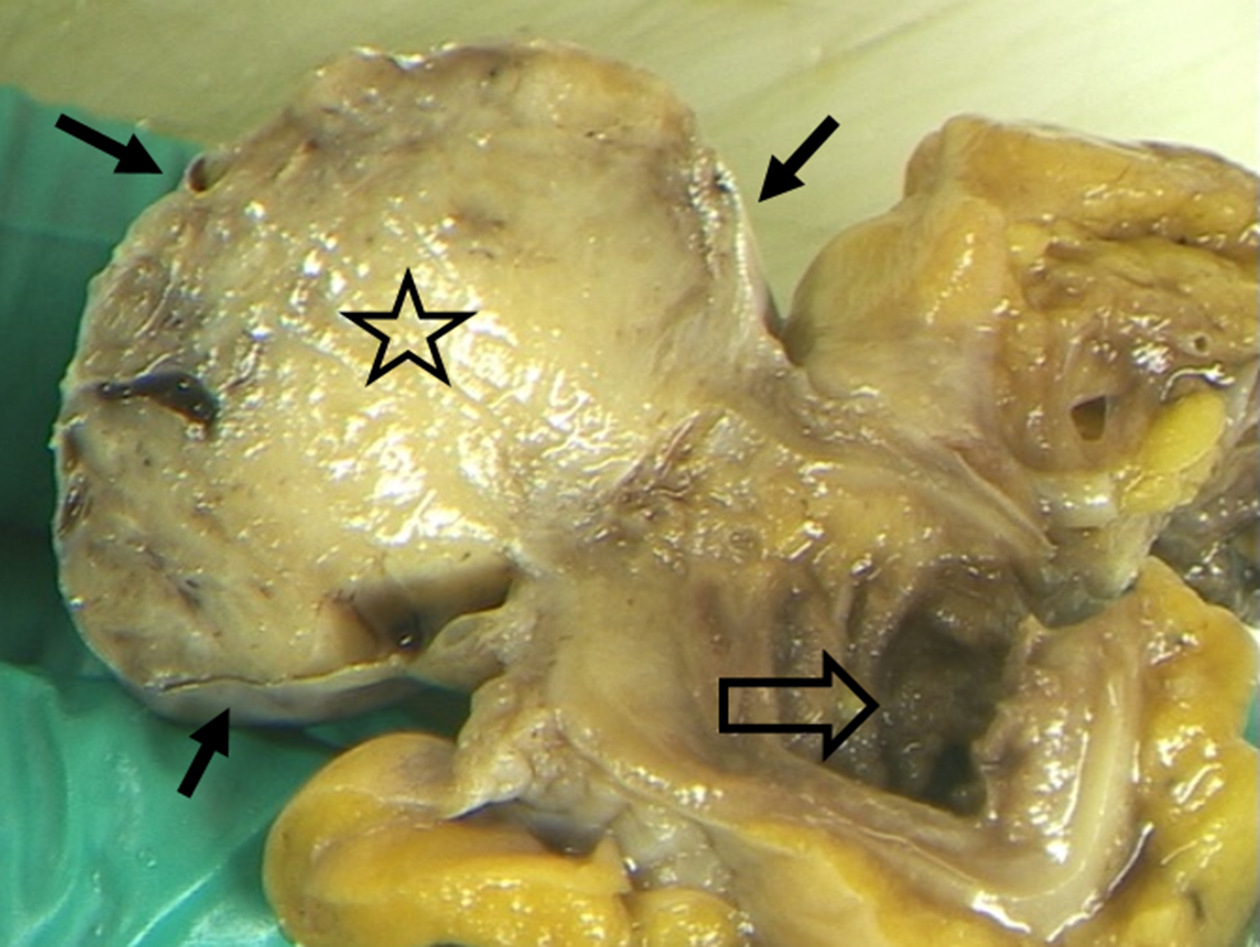
Gross pathology. Cut on exophytic greyish-white to blue gray tumour mass (star), encapsulated by serosa (small arrows), protruding from the terminal ileum (large arrow), which is characterized by small valvulae conniventes, also known as Kerckring folds or plicae circulares. On the top right the cecum begins.

**Figure 8.**
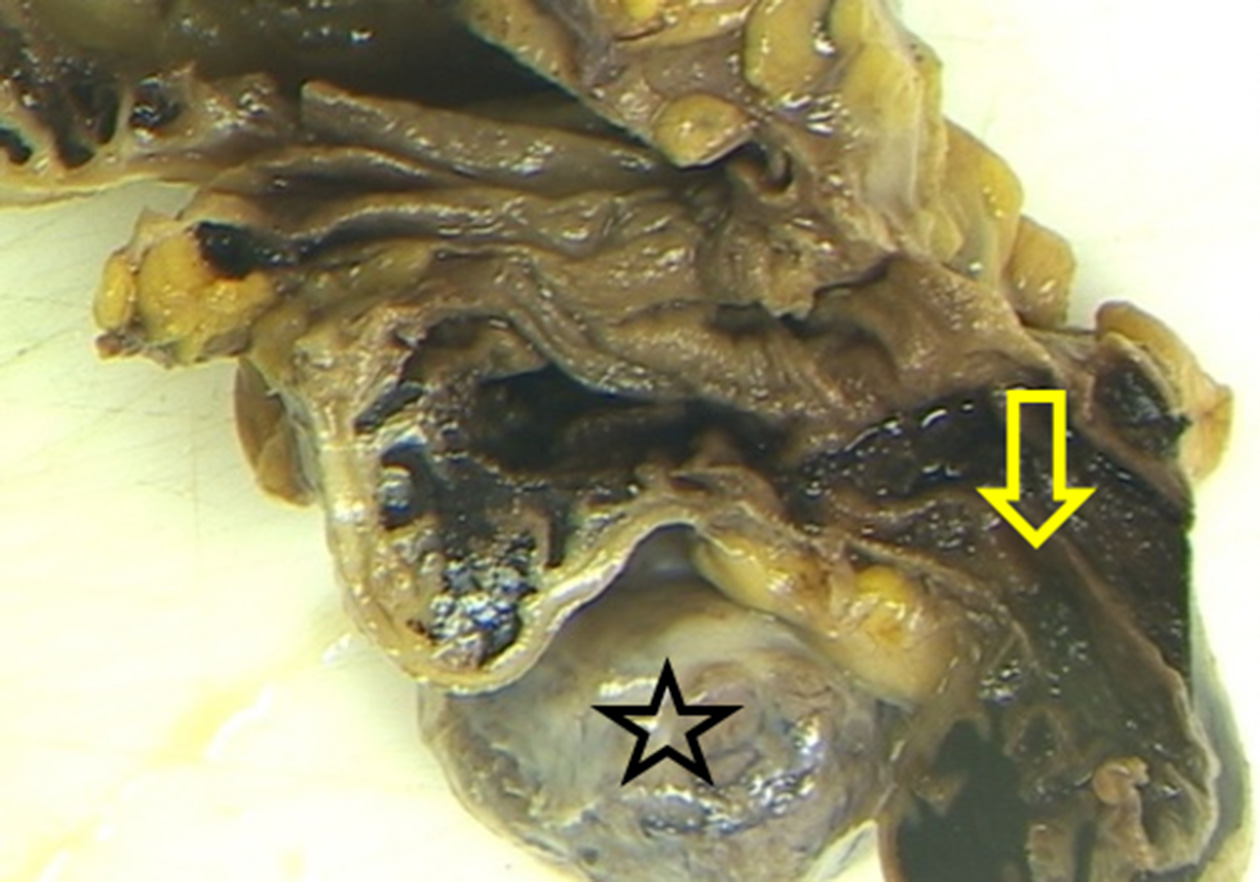
Gross pathology. Extensive blood clots intraluminally in the cecum and ileum (yellow arrow). Exophytic ileal bluish-gray tumour mass (star).

On histopathology, a greyish-white to blue grey tumour mass of approximately 9  cm was seen, encapsulated by serosa with a solid nodular, fibrous aspect that was hard on palpation (Figure 7). The tumour process was resected marginally and was located subserosal and submucosal with a growth of about 55% circumferentially. The mucosa shows no abnormalities. There was a hypertrophic vessel overlying the tumour mass and the lumen of the resected intestine was filled with blood clots and the bowel showed signs of dilatation (Figure 8). The appendix was normal. Twelve reactive lymph nodes were found.

**Figure 9.**
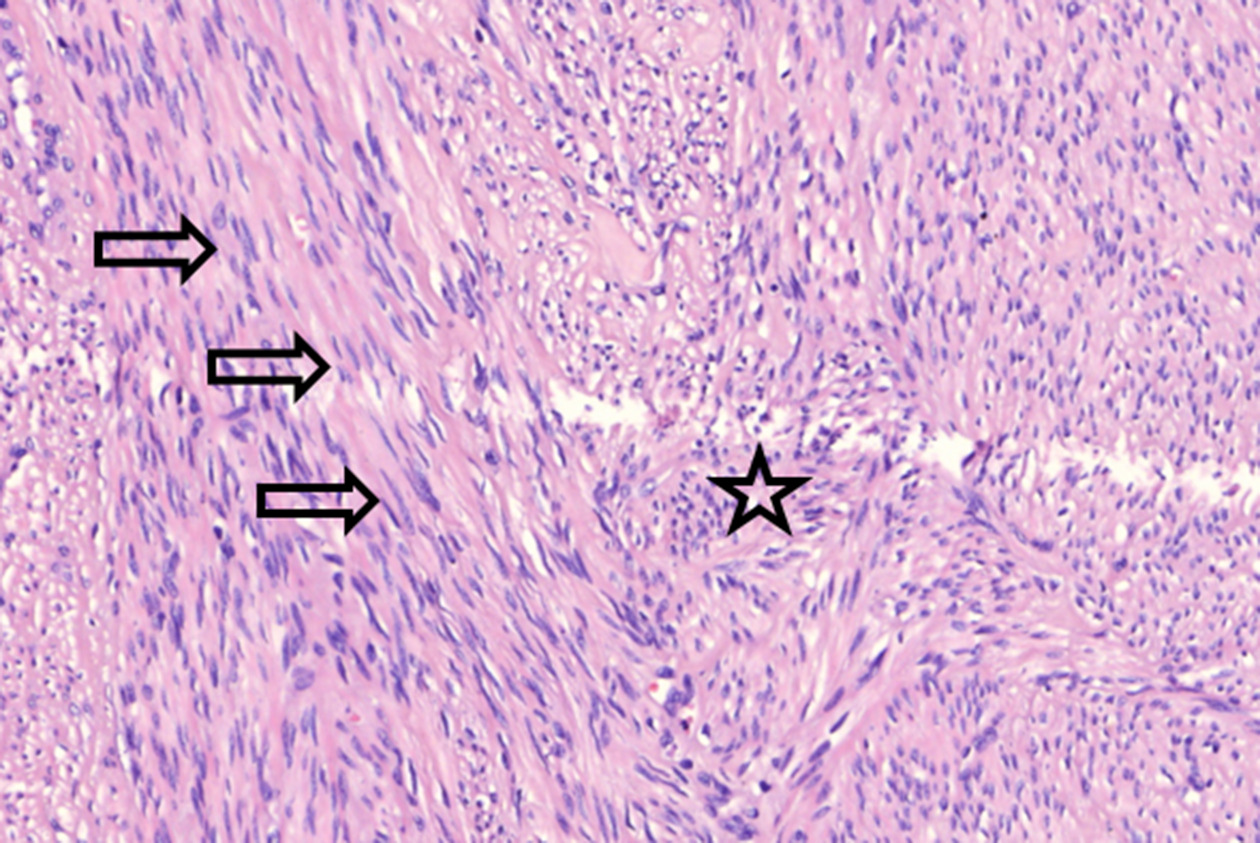
Hematixylin and eosin stained, 400× magnification. Cell rich tumour proliferation with spindle- or sigar-shaped elongated nuclei (arrows) in a palisaded or storiform pattern of long and criss-cross intersecting bundles (star). Limited non-typical mitotic figures, 5–10 per 10 high power fields (not on this slide).

**Figure 10.**
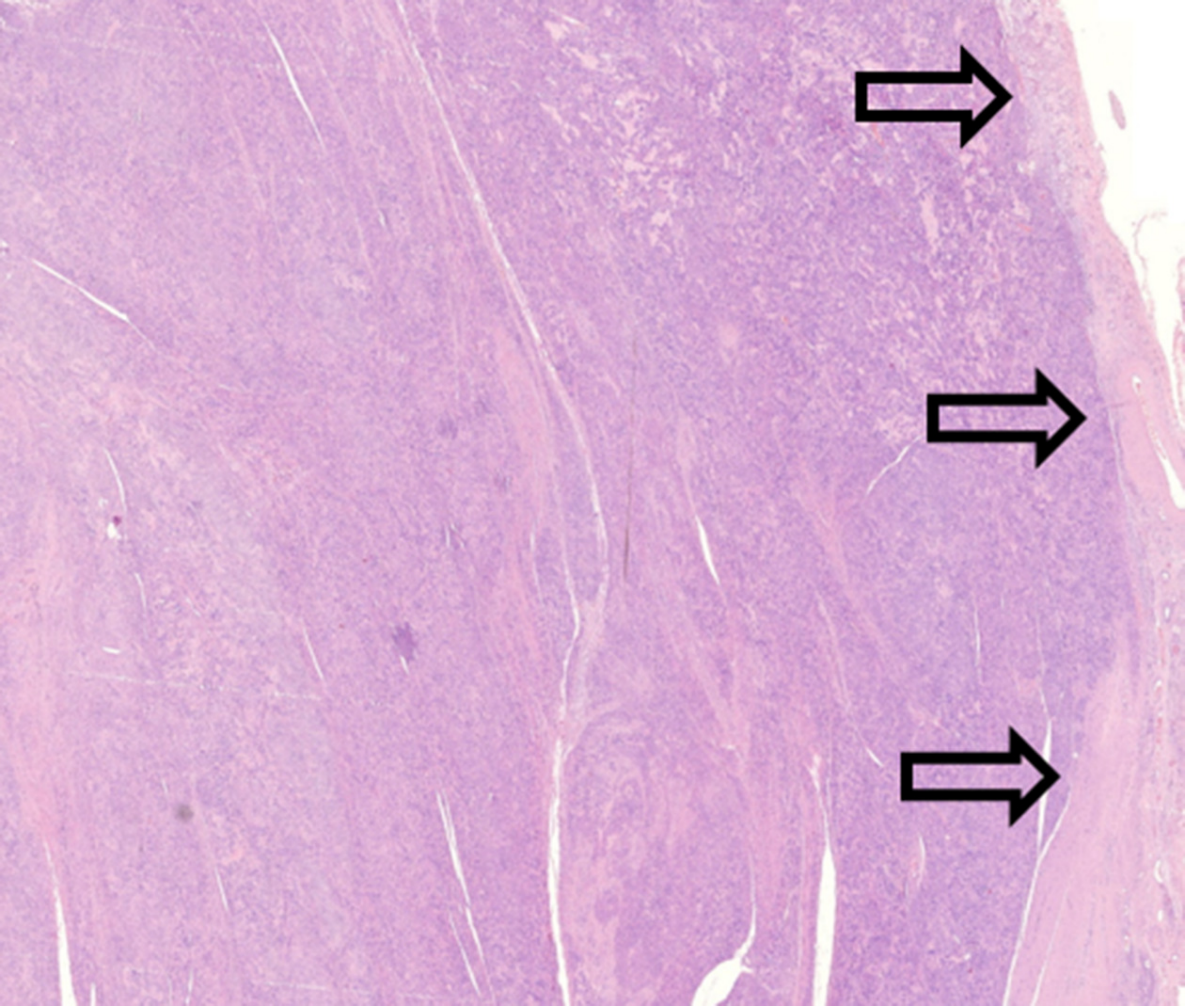
Hematoxylin and eosin stained, 25× magnification. Cell rich lesion, lying in the muscularis propria layers, encapsulated by serosa (arrows).

**Figure 11.**
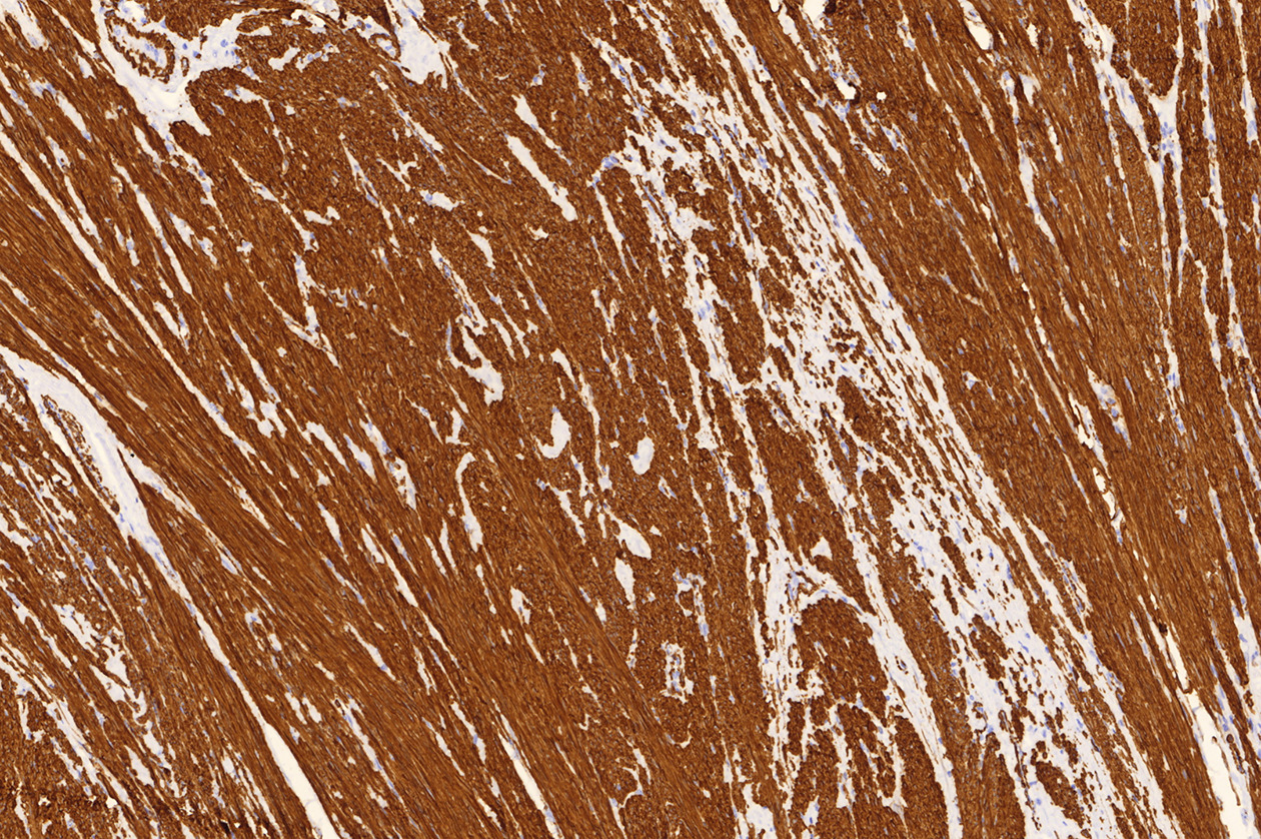
SMA stained, 400× magnification. SMA positive (brown stained) tumour cells, indicating presence of smooth muscle cells, an important feature for differentiating leiomyosarcoma from gastrointestinal stromal tumour. SMA, smooth muscle actin.

**Figure 12.**
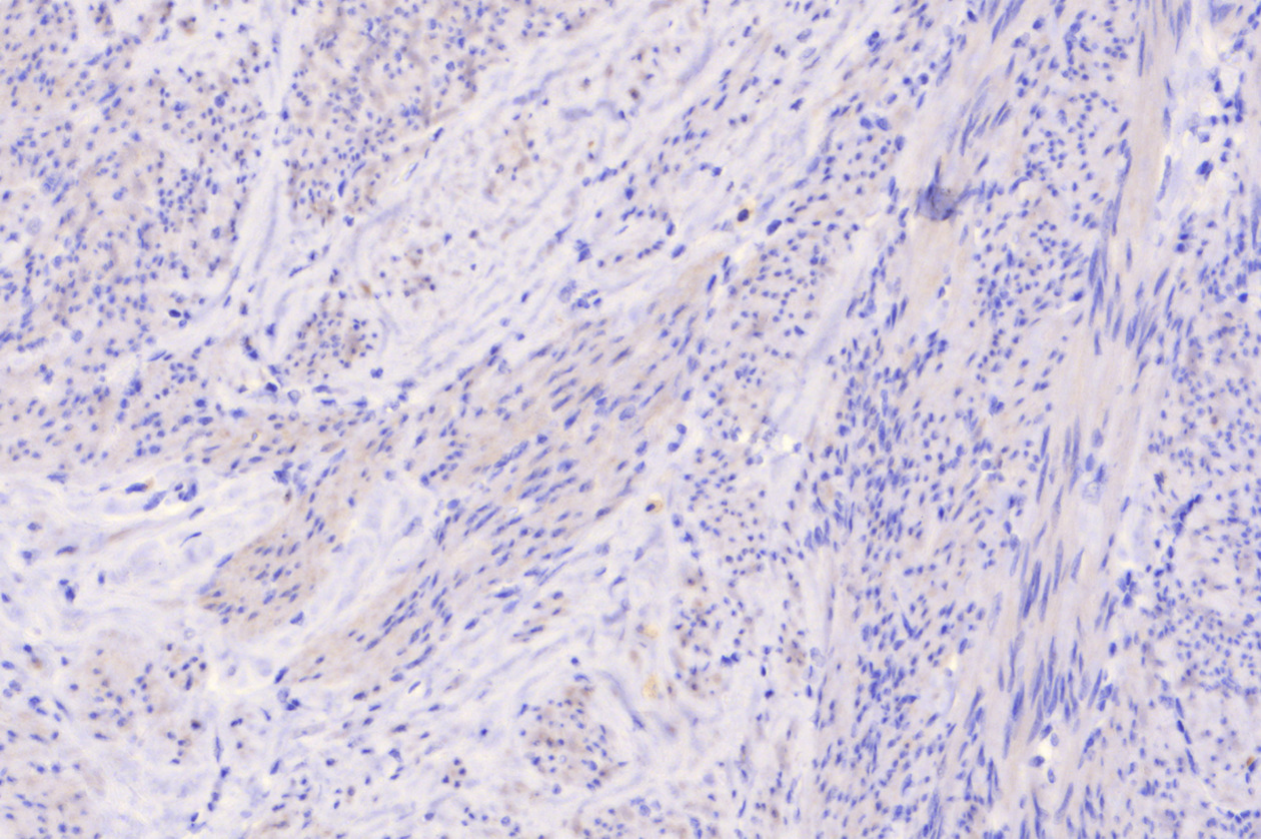
KIT (CD 117) stained, 400× magnification. Spindle-shaped cores of nuclei with no positive staining (cells are not coloured brownish) on KIT, thereby excluding gastrointestinal stromal tumour.

Microscopically, a cell rich lesion was seen, composed of spindle-shaped cells that were arranged in a palisaded, or fascicular growth pattern with “cigar-shaped” nuclei, embedded in eosinophilic cytoplasm (Figures 9 and 10). The cores contained fine chromatin with some variation in core size. The cytonuclear atypia was limited with some mitotic figures. There were no large, bizarre nuclei nor necrosis. Immunohistochemical analysis showed diffuse strong expression of the tumour cells for caldesmon, SMA and desmin, corresponding with a myogenic differentiation (Figure 11). The tumour cells were negative for proto-oncogene receptor tyrosine kinase (KIT) or CD117, CD34, S100 and DOG 1 stains, as well as for oestrogen and progesterone receptors, all markers that are positive in gastrointestinal stromal tumours (Figure 12). MIB-1 showed a proliferation fraction of less than 5%.

In summary, it concerned a mesenchymal spindle-shaped lesion with myogenic differentiation and a slight degree of cytonuclear atypia with some mitotic figures. The pathological differential diagnosis was an intramural leiomyoma (LM) or a low-grade leiomyosarcoma (LMS). Intramural leiomyomas (LMs) of the digestive tract occur; however, they show no cytonuclear atypia and they are rarely larger than 2 cm in diameter, as described in the World Health Origanisation classification of intestinal tumours.^1^ Given the large size of this tumour (9  cm), the slight cytonuclear atypia and some mitotic figures that are found, there was a preference to classify the tumour as low-grade LMS of the gastrointestinal (GI) tract over an intramural LM. A GI stromal tumour (GIST) was excluded immunohistochemically. High grade LMS would be characterized by areas of necrosis and haemorrhage, large bizarre nuclei and a large proliferation rate or multiple abnormal mitoses.^1^

## Differential diagnosis

The main radiological differential diagnosis of LMS is GIST, which morphologically resembles LMS. Both LMS and GIST can either grow exophytic (50%) or intraluminal,^1,2^ although GIST has a larger propensity for intraluminal growth than LMS. On CT, both LMS and GIST are large, lobulated, exophytic masses with heterogeneous enhancement and sometimes dystrophic tumoural calcifications.^3^ LMS tends to be somewhat more homogeneous and hypodense on non-enhanced CT scan. Both LMS and GIST can have cystic or necrotic components, depending on the malignancy grade and size.^3,4^ Both tumours can potentially to ulcerate and cause GI bleeding when ulceration reaches the mucosal surface. On MRI scan, LMS is hypointense on *T*_1_ weighted images, intermediate of signal intensity on *T*_2_ weighted images and shows heterogenous contrast-enhancement with central necrotic areas.^5^ Both GIST and LMS may metastasize to liver and peritoneum by direct invasion, peritoneal or haematological spread.^3,4^ Of all GIST, 50% present with liver or peritoneal metastases.^4^ Typically, GIST does not display lymphatic spread, but LMS does.^6^

On both macroscopy and microscopy LMS and GIST share the same features: both are mesenchymal tumours embedded in the muscle layers (muscularis propria) that consist of spindle cells, with a myogenic differentiation, arranged in a palisaded or storiform pattern with pale eosinophilic indistinct cytoplasm. Therefore, immunohistochemistry plays an important role to differentiate LMS from GIST, since these two tumours resemble each other on gross macroscopy. Approximately 85% of GISTs harbour activating mutations in the KIT or platelet derived growth factor receptor alpha (*PDGFRA*) gene. Moreover, approximately 95% of GISTs are positive for CD117 by immunohistochemistry.^1,2^ LMSs are differentiated from GISTs by the lack of CD117 (c-KIT), DOG1 and CD34 as well as presence of smooth muscle actin (SMA) and desmin.^2^

The second differential diagnosis are LMs, common benign neoplasms of the small bowel. While LMSs are mostly located in the jejunum, LMs can occur anywhere in the small bowel, but mainly in the ileum and jejunum.^3^ LMs are typically 1–2 cm in size, rounded and well-circumscribed but as LMSs they can ulcerate and cause bleeding.^4^ LM are hypervascular on angiography and show a more moderate, homogeneous enhancement in comparison to LMS. On MRI scan, leiomyomas have an intermediate *T*_1_ signal and slightly increased *T*_2_ signal.^7^ Low grade LMS may show more irregular lesion margins and present with regional lymphadenopathy. Leiomyosarcomas are often larger (>6 cm), but both LM and LMS can have necrosis and calcifications.^5^ Their imaging findings are comparable and there are no definite features to preoperatively distinguish low grade LMS from LMs on imaging.

## Discussion

Most tumours of the small bowel are metastases. Primary small bowel tumours are rare, and account for less than 5% of all GI malignancies. Of these, the majority are adenocarcinomas (40%) and carcinoids (31%), followed by lymphomas (20%) and mesenchymal tumours (9%), among which GIST and sarcomas, showing an increasing incidence over the years, partly caused by various associated genetic disorders.^8^ LMS represents 2–3% of small bowel tumours.^8^ The incidence is estimated 22.7 cases per million and less than 30 case reports worldwide have been found in literature.^6,8^

Patients present between age 50 and 70 with a small male preponderance.^6^ The most frequent origin is the jejunum, followed by ileum and duodenum, respectively. Most small bowel LMS grow towards the subserosal side of the bowel (66%), without an intraluminal component.^1^ Therefore, patients remain asymptomatic in the early stages of disease. Later, they can present with ferriprive anaemia or chronic intermittent melena, abdominal pain, a palpable mass or intussusception (especially ileal LMS).^6,9^ Because of its aspecific or asymptomatic clinical presentation and the difficulty to visualize the small bowel by upper and lower endoscopy, LMS is discovered at advanced stage.^9^ Moreover, since LMS grows mainly subserosal, it can easily be missed since endoscopy only covers intraluminal inspection.

Preoperative diagnosis of small intestine tumours remains difficult, especially distinguishing benign and malignant tumours.^6^ CT scan has the advantages of low cost and fast imaging. In acute setting, CTA has a high sensitivity between 86% and 95% for detecting the source of GI bleeding.^10^ In contrary, endoscopy in acute setting suffers from disadvantages as inadequate bowel preparation, low sensitivity of 20–40% ^10^ and risks of sedation, perforation and bleeding.^10^ MRI scan offers best soft tissue contrast and may be more sensitive to detect small mucosal lesions, plus it can differentiate tumours based on their signal characteristics without the need for ionisation.^6^

Currently, additional techniques such as MRI enterography, MRI enteroclysis and wireless capsule endoscopy (WCE) have shown to detect mucosal abnormalities and thus to diagnose small bowel tumours.^7,9^ However, their use may be limited in the setting of acute GI blood loss or obstruction and their sensitivity is low in evaluation of mural and extramural neoplasms.^5^ Another important limitation of wireless CE is capsule retention in approximately 10–25% of cases, leading to the urge of acute surgery because of acute bowel obstruction and sometimes perforation.^5^ In patients with suspected small bowel tumours, MRI enteroclysis might be used as the first modality of choice, followed by double-balloon endoscopy for histological determination.^5^ Finally, positron emission tomography can contribute in evaluating metastases, but imaging results depend on histological grade of the tumour and its metabolism.^11^ Therefore low grade LMS might not show uptake of radioactive labelled glucose.^11^

## Treatment

Treatment is primarily surgical resection.^9^ Preoperative embolisation plays no significant role. Radical resection can be curative, unless there are already peritoneal or liver metastases. While adjuvant chemotherapy is used for GIST and uterine LMS, little efficacy has been reported for small bowel LMS, neither for radiation therapy.^12^

## Prognosis

The most important prognostic factor is malignancy grade (FNCLCC or Trojani), determined by differentiation grade (I–III), the number of mitoses per 10 high power field (0–9, 10–19 and >20) and necrosis (none, <50%, >50%)^2^ Second, histological predictors are tumour size, mucosal invasion and cellular atypia.^1,2^ Finally, the presence of liver and peritoneal metastases (estimated 24–50%) predicts the 5-year disease free survival, which between 28% and 48%.^12^ The overall prognosis of small bowel LMSs is better than adenocarcinomas, but it remains poor.

## Conclusions

LMS is a rare small bowel malignancy that infrequently can present with acute GI bleeding. In emergency setting, CTA has a high sensitivity to detect this large, mainly exophytic mass of the small intestine. Accurate management relies on a surgical approach. Given the relatively inaccessible location of the small intestine to both upper and lower GI endoscopy, this case highlights the importance of imaging and the role for the radiologist when faced with a differential diagnosis of small bowel malignancy. The pathologist makes the final call in differentiating LMS from the much more common GIST.

## Learning points

1. Primary LMS of the small bowel is a rare GI malignancy and can present with acute GI bleeding.
2. Immunohistochemistry plays a key role in differentiating LMSs from GIST, since they are hardly indistinguishable on imaging.
3. When small bowel malignancy is suspected in a patient and upper and lower endoscopies are normal, CTA is the imaging of choice which also has a high sensitivity. In outpatients, diagnostic work-up should include MRI enteroclysis, MRI enterography or wireless capsule endoscopy.

## Consent

Informed consent to publish (including images and data) was obtained and is held on record.
